# Management of incessant ventricular arrhythmias in a patient with left ventricular assist device: a case report

**DOI:** 10.1186/s13019-024-02659-1

**Published:** 2024-04-01

**Authors:** Chen Chen, Juan Du, Xianqiang Wang, Liang Zou

**Affiliations:** 1 Department of Cardiovascular Surgery, Fuwai Hospital, National Center for Cardiovascular Diseases, Chinese Academy of Medical Sciences and Peking Union Medical College, No. 167 Beilishi Road, Xicheng District, Beijing, 100037 People’s Republic of China; 2 National Health Commission Key Laboratory of Cardiovascular Regenerative Medicine, Fuwai Central-China Hospital, Central-China Branch of National Center for Cardiovascular Diseases, No.1 Fuwai Avenue, Zhengdong New District, Zhengzhou, 451464 People’s Republic of China

**Keywords:** Ventricular arrhythmias, Left ventricular assist device, Heart failure, Heart transplantation

## Abstract

**Background:**

The implantation of left ventricular assist devices (LVADs) as a bridge to transplantation or as destination therapy in end-stage heart failure patients is frequently complicated by the emergence of ventricular arrhythmias (VAs). These arrhythmias have been implicated in precipitating deleterious clinical outcomes, increased mortality rates and augmented healthcare expenditures.

**Case Presentation:**

We present a challenging case of a 49-year-old male with a history of dilated cardiomyopathy who received an LVAD. Post-implantation, the patient suffered from intractable VAs, leading to multiple rehospitalizations and hemodynamic deterioration. Despite exhaustive medical management and electrical cardioversion attempts, the patient’s VAs persisted, ultimately necessitating prioritization for cardiac transplantation.

**Discussion:**

This case highlights the challenges in managing VAs in LVAD patients and the importance of multidisciplinary collaboration. While pharmacological intervention is the initial strategy, catheter ablation may be considered in selected cases when medication is insufficient. In instances of intractable VAs, expeditious listing for heart transplantation as a high-priority candidate is advisable when feasible.

## Background

Heart failure (HF) has steadily risen worldwide over the last decade. An estimated 13.7 million individuals in China have HF, and 2.7% of HF patients are graded as having moderate or severe left ventricular diastolic dysfunction [1]. LVAD has become an effective therapeutic approach for treating patients with refractory heart failure when acting as a bridge to transplantation or destination therapy in patients ineligible for heart transplantation [2].

It is widely known that LVAD implantation is associated with an increased risk of VAs. VAs are defined as sustained (> 30 s) ventricular tachycardia (VT) or ventricular fibrillation (VF) occurring after LVAD implantation without an acute reversible cause and requiring effective termination by external electrical shock or medical therapy [3]. The current literature reports that incidence of VAs after LVAD implantation ranges from 28–49% [4–6]. Ventricular arrhythmias can be well-tolerated with LVAD but if persistent, they may lead to haemodynamic compromise, right ventricular (RV) failure, and secondary organ dysfunctio [7, 8]. The management of ventricular arrhythmias in LVAD patients remains challenging and requires close collaboration of heart failure cardiologists, arrhythmiologists, and heart transplant surgeons.

We present a case involving a patient equipped with an LVAD but without an ICD (Implantable Cardioverter Defibrillator), who manifested persistent and intractable ventricular arrhythmias, ultimately necessitating heart transplantation.

### Case presentation

In December 2020, a 49-year-old male patient with a documented history of dilated cardiomyopathy presented to Fuwai Hospital. The patient reported experiencing intermittent dyspnea for over ten years, which had intensified in the past year. Upon admission, he was diagnosed with dilated cardiomyopathy, arrhythmia, persistent atrial fibrillation, ventricular tachycardia, advanced heart failure (NYHA class IV), and type 2 diabetes mellitus. Traditional pharmacological interventions yielded limited therapeutic response; therefore, he underwent successful implantation of a left ventricular assist device (LVAD, model: Corheart6) as destination therapy in January 2022. Impressively, his functional status was upgraded to NYHA class II, and he demonstrated the capacity to ambulate 500 m within six minutes, supported by an LVAD setting of 3200 rpm, ensuring a flow rate of 4.1 L/min. After 51 postoperative days, he was considered stable for discharge.

Nonetheless, in July 2022(6 months post-operation), he was readmitted after experiencing three days of diarrhea and palpitations. On examination, the patient appeared alert but fatigued. Hemodynamic assessment revealed mean blood pressure was 65 mmHg at LVAD pump speed of 3100 rpm, and the estimated flow was 2.8 L/ min. Laboratory analyses highlighted increased serum creatinine (150.2 umol/l) and elevated levels of N-terminal prohormone of brain natriuretic peptide (7223 pg/mL). All other laboratory tests are within normal limits. A 24-hour Holter monitor identified episodes of paroxysmal atrial fibrillation and ventricular tachycardia, with the most prolonged episode spanning 4333 beats. Transthoracic echocardiography disclosed a severely dilated left ventricle (end-diastolic diameter: 79 mm) with a significantly depressed ejection fraction of 20%. Throughout the evaluation, the aortic valve remained closed.

**Fig. 1:**
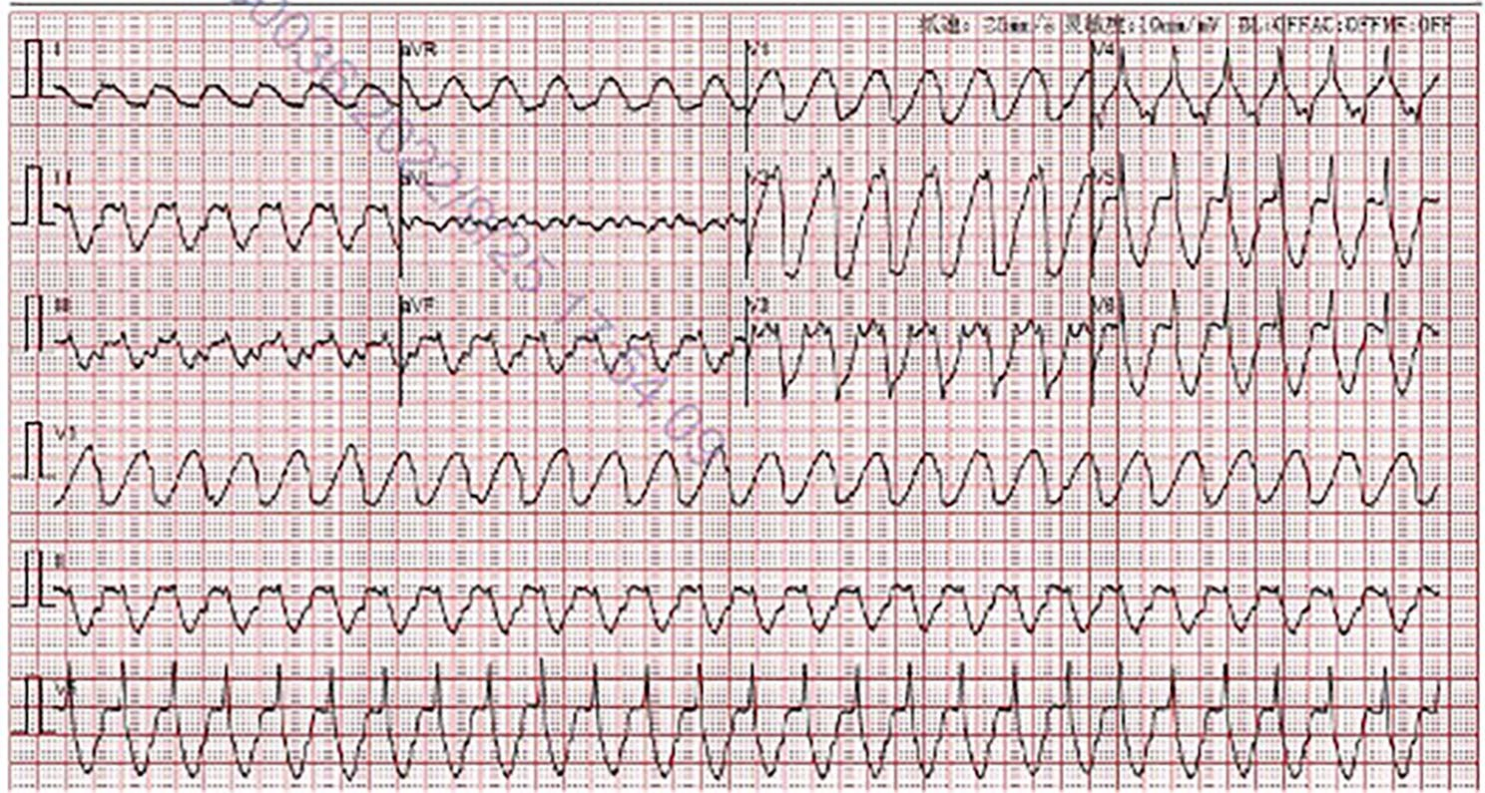
Electrocardiogram demonstrating ventricular tachycardia

**Fig. 2:**
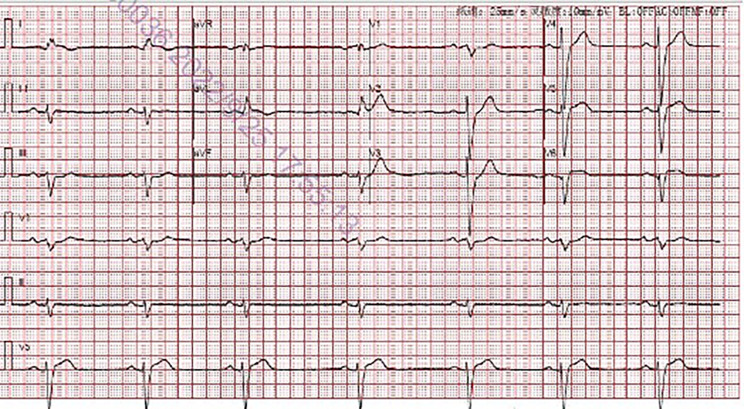
Electrocardiogram after therapy demonstrating sinus bradycardia

On the evening of his readmission, the patient underwent an electrical storm, characterized by repeated ventricular tachycardia (Fig. 1). Immediate electrical cardioversion was initiated after echocardiographic exclusion of intracardiac thrombi. However, arrhythmic recurrences persisted, warranting combination therapy with amiodarone, esmolol, lidocaine, and magnesium sulfate, which eventually reestablished regular rhythm within four hours. Given the post-defibrillation sinus rhythm’s latency (Fig. 2) and the contraindications associated with certain antiarrhythmics, a provisional RV apical pacemaker was implanted. The heightened excitability of the myocardium rendered ventricular arrhythmias challenging to rectify. Consequently, a regimen of oral amiodarone and bisoprolol was initiated, effectively managing the arrhythmic episodes. The patient was discharged two weeks post-intervention.

**Fig. 3:**
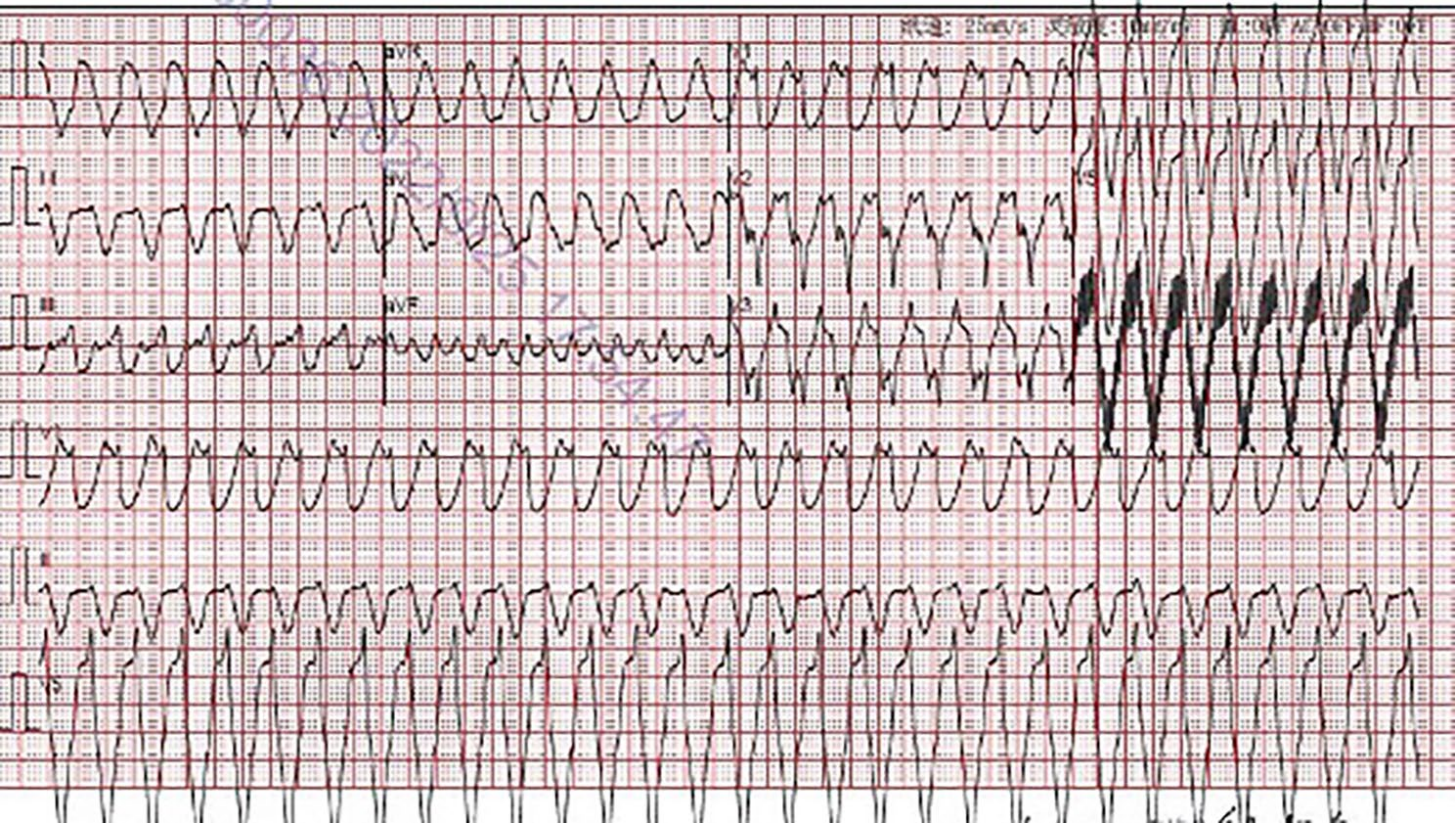
Electrocardiogram demonstrating ventricular tachycardia

**Fig. 4:**
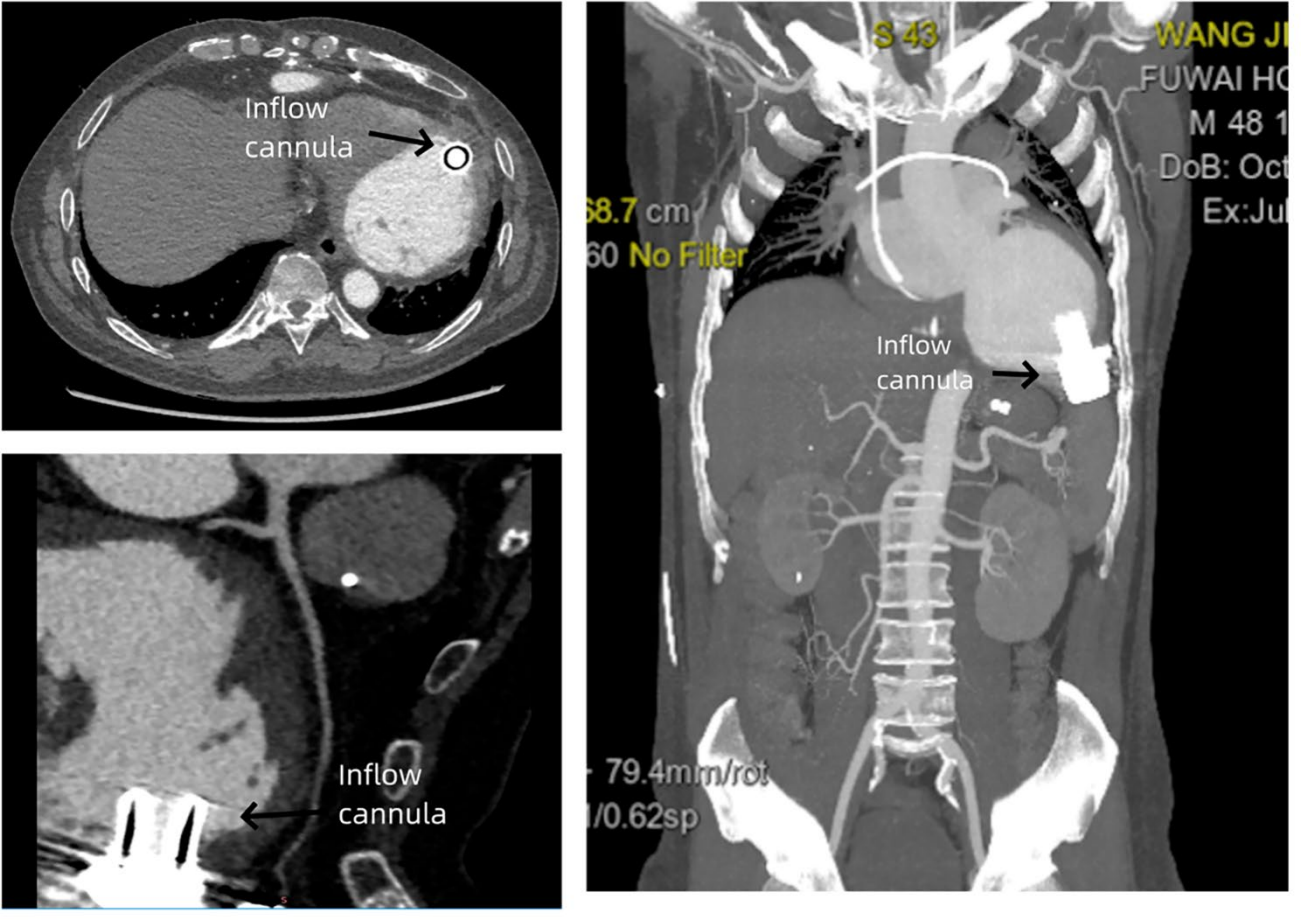
Cardiac computed tomography showing an appropriately positioned left ventricular assist device inflow cannula

In August 2022 (7 months post-operation), the patient returned, reporting frequent low-flow alarms from the LVAD. He described symptoms of thoracic discomfort and dizziness. Preliminary evaluation indicated a heart rate of 200 beats/min, mean arterial pressure of 80 mmHg with the LVAD at 3200 rpm, an estimated flow rate of 1.7 L/min, oxygen saturation at 93%, and a respiratory rate of 25 breaths/min. Driveline site inspection revealed no infectious signs. An electrocardiography identified ventricular tachycardia (Fig. 3), persisting even after defibrillation. Due to recurrent ventricular tachycardia episodes, the LVAD speed was reduced from 3200 rpm to 3100 rpm, achieving a flow rate of 2.6–2.85 L/min. LVAD rotation was adjusted to the minimal level necessary for efficient left ventricular offloading. The electrocardiography suggested potential origins of ventricular tachycardia from the left ventricular basal wall, raising suspicions about the LVAD’s potential role. A cardiac computed tomography scan authenticated the standard mechanical alignment between the left ventricular septal wall and the LVAD inflow cannula, negating any potential sources of ectopic rhythms (Fig. 4).

Faced with recurrent life-threatening arrhythmias and the patient’s deteriorating condition, the clinical team reached a consensus on the need for a definitive solution. Given the elevated risks associated with catheter ablation, given the patient’s compromised state, prioritization for a heart transplant was deemed essential. In September 2022, the patient underwent a successful heart transplantation. The surgical procedure was devoid of complications, and postoperative ventricular arrhythmias were absent, culminating in an uneventful discharge.

## Discussion and conclusions

In this study, we utilize a case of persistent VAs following LVAD placement to elucidate LVAD physiology, explore resuscitation strategies for acutely decompensated patients, and furnish a contemporary overview of antidysrhythmic medications intended for the cessation of ventricular dysrhythmias. Post-LVAD implantation, VAs emerge as a common complication. Incidences of late-onset ventricular arrhythmias, occurring > 30 days post-implantation, were noted in 27% of patients, with a median follow-up duration of 19 months. Furthermore, an electrical storm was observed in 9% of this cohort [9]. VAs have the potential to compromise the hemodynamic efficacy of the LVAD, leading to increased hospital admissions and heightened demand for antiarrhythmic interventions, external defibrillations, and urgent heart transplants [10, 11]. The etiology of post-implantation VAs is multifaceted, encompassing factors such as suction events, electrical remodeling, pre-existing myocardial scars, and mechanical irritation from the LVAD cannula [12, 13].

The literature does not offer a unanimous approach to managing LVAD patients presenting with such dysrhythmias. It’s imperative to ascertain and address the arrhythmic trigger. Potential triggers, ranging from electrolyte imbalances to acute ischemia, fever, underlying illness, suction events, and ventricular irritation due to inflow cannula contact, necessitate prompt management. Initial therapeutic interventions should prioritize pharmacological measures, such as antiarrhythmic drugs or cardioversion. However, current literature [14–16]does not demonstrate the superiority of any agent for VAs in terms of survival or neurologic outcome. A slight benefit of amiodarone over lidocaine was observed in witnessed out-of-hospital arrests with effective bystander cardiopulmonary resuscitation [17]. This finding may be applicable to LVAD patients, given the device’s ability to maintain circulation without an organized rhythm. Notably, while amiodarone has broad antidysrhythmic effects, other agents might specifically VAs [18]. In scenarios of recurrent VAs unresponsive to antiarrhythmic therapy, catheter ablation emerges as a viable alternative, requiring skilled operators versed in LVAD physiology and collaborative efforts between heart failure cardiologists and arrhythmiologists [19, 20]. If catheter ablation is impractical or deemed too risky due to severe comorbidities, including chronic driveline infections or hemodynamic intolerance, stellate ganglion ablation could be envisioned as a last-resort approach for patients with end-stage heart failure undergoing LVAD therapy who are plagued by relentless VAs. The justification and use of stellate ganglion ablation typically emerge in cases where conventional treatment methods, such as antiarrhythmic drugs and catheter ablation, have either been ineffective or are considered inappropriate. A representative example [21] is a 72-year-old patient with an LVAD implant, who experienced persistent VAs resistant to both antiarrhythmic medication and catheter ablation. To address this, a video-assisted thoracoscopic sympathectomy was undertaken, providing significant symptomatic relief for a period of 150 days thereafter.

Suction events arise when there is inadequate preload to the LV. Such events are more readily identifiable in the context of hypovolemia, where the LV preload is insufficient, leading the inflow cannula to be obstructed by the myocardial wall. In such instances, fluid resuscitation and an assessment of the underlying cause of the suction event are warranted. Conversely, hypervolemia or other etiologies of right heart failure can precipitate suction events when the RV inadequately supplies preload to the LV. Ultrasound and cardiac computed tomography are valuable in evaluating ventricular dimensions, functionality, overall fluid status, and the position of the inflow cannula [18]. In this case, the left ventricle was dilated, with the inflow cannula optimally positioned.

This case underscores the feasibility of sustaining hemodynamic stability amidst prolonged VAs in patients supported by LVAD. Extant literature [22] underscores the ‘Fontan-like circulation’ observed in patients with VAs and LVAD, capable of maintaining optimal hemodynamics. With the LVAD’s support, such patients can tolerate life-threatening VAs for extended periods. As the left ventricle undergoes unloading, a marked decline in pulmonary resistance ensues, enhancing pulmonary circulation. During this juncture, the right ventricle predominantly serves as a conduit. Although VAs in LVAD-supported patients are usually tolerated, extended episodes may precipitate left ventricular collapse, resulting in hemodynamic instability or RV failure. Managing LVAD patients exhibiting ventricular arrhythmias resulting in exacerbated RV failure presents a formidable challenge. In instances of RV dysfunction or hemodynamic collapse, prompt hemodynamic support is imperative to prevent irreversible end-organ damage. There is a parallel case [7] involving a 54-year-old patient with dilated non-ischemic cardiomyopathy, sustained by LVAD (HeartMate3) as a bridging strategy for transplantation. This patient remained stable for 35 days, experiencing persistent VAs and RV failure, managed concomitantly with VA-ECMO and LVAD, culminating in heart transplantation. For patients confronting intractable ventricular arrhythmias under LVAD support, heart transplantation remains the gold standard [23]. Thus, assessing these patients for transplant eligibility is crucial, ensuring no contraindications preclude the procedure and minimizing secondary organ damage.

In the realm of the management of ventricular arrhythmias in LVAD patients, prophylactic strategies are of utmost significance. Contemporary research [24] highlights the fact that a prior history of VAs is the major independent predictor of post-operative arrhythmia. Consequently, a comprehensive preoperative evaluation of cardiac arrhythmia history is imperative in patients scheduled for LVAD implantation. In individuals identified as high-risk, the pre-surgical assessment should incorporate considerations for the deployment of an ICD. Furthermore, vigilant monitoring and management of postoperative electrolyte imbalances are essential, with a particular focus on augmenting magnesium and potassium levels. The judicious application of antiarrhythmic agents such as amiodarone and bisoprolol is also recommended to attenuate myocardial excitability, thereby mitigating the risk of post-operative arrhythmia.

In summary, VAs are prevalent in patients with LVADs and are generally well-tolerated unless hemodynamic compromise ensues. Identifying and addressing the arrhythmic trigger is paramount, with ablation as a consideration for stable patients. If hemodynamic compromise occur, heart transplantation is highly recommended.

## Abbreviations

LVAD: Left ventricular assist device
VAs: Ventricular arrhythmias
HF: Heart failure
VT: Ventricular tachycardia
VF: Ventricular fibrillation
RV: Right ventricular
ICD: Implantable Cardioverter Defibrillator
BP: Blood Pressure
HR: Heart Rate
PAP: Pulmonary Arterial Pressure
PAWP: Pulmonary Artery Wedge Pressure
CO: Cardiac Output
CI: Cardiac Index
SVR: Systemic Vascular Resistance
PVR: Pulmonary Vascular Resistance
RVSWI: Right Ventricular Stroke Work Index
CVP: Central Venous Pressure
